# Pupillometric analysis for assessment of gene therapy in Leber Congenital Amaurosis patients

**DOI:** 10.1186/1475-925X-11-40

**Published:** 2012-07-19

**Authors:** Paolo Melillo, Leandro Pecchia, Francesco Testa, Settimio Rossi, Jean Bennett, Francesca Simonelli

**Affiliations:** 1 Department of Ophthalmology, Second University of Naples, Naples, Italy; 2 Department of Electronics, Computer Science and Systems, University of Bologna, Bologna, Italy; 3 Department of Electrical & Electronic Engineering, University of Nottingham, Nottingham, United Kingdom; 4 Center for Cellular and Molecular Therapeutics, Children’s Hospital of Philadelphia (CHOP), Philadelphia, PA, USA; 5 F.M. Kirby Center for Molecular Ophthalmology, University of Pennsylvania, Philadelphia, PA, USA

## Abstract

**Background:**

Objective techniques to assess the amelioration of vision in patients with impaired visual function are needed to standardize efficacy assessment in gene therapy trials for ocular diseases. Pupillometry has been investigated in several diseases in order to provide objective information about the visual reflex pathway and has been adopted to quantify visual impairment in patients with Leber Congenital Amaurosis (LCA). In this paper, we describe detailed methods of pupillometric analysis and a case study on three Italian patients affected by Leber Congenital Amaurosis (LCA) involved in a gene therapy clinical trial at two follow-up time-points: 1 year and 3 years after therapy administration.

**Methods:**

Pupillary light reflexes (PLR) were measured in patients who had received a unilateral subretinal injection in a clinical gene therapy trial. Pupil images were recorded simultaneously in both eyes with a commercial pupillometer and related software. A program was generated with MATLAB software in order to enable enhanced pupil detection with revision of the acquired images (correcting aberrations due to the inability of these severely visually impaired patients to fixate), and computation of the pupillometric parameters for each stimulus. Pupil detection was performed through Hough Transform and a non-parametric paired statistical test was adopted for comparison.

**Results:**

The developed program provided correct pupil detection also for frames in which the pupil is not totally visible. Moreover, it provided an automatic computation of the pupillometric parameters for each stimulus and enabled semi-automatic revision of computerized detection, eliminating the need for the user to manually check frame by frame. With reference to the case study, the amplitude of pupillary constriction and the constriction velocity were increased in the right (treated eye) compared to the left (untreated) eye at both follow-up time-points, showing stability of the improved PLR in the treated eye.

**Conclusions:**

Our method streamlined the pupillometric analyses and allowed rapid statistical analysis of a range of parameters associated with PLR. The results confirm that pupillometry is a useful objective measure for the assessment of therapeutic effect of gene therapy in patients with LCA.

**Trial registration:**

ClinicalTrials.gov NCT00516477

## Background

Leber congenital amaurosis (LCA) is a rare ocular disease, affecting around 1 in 81,000 people [1], and is one of the most severe forms of inherited retinal degeneration. LCA patients have severe loss of vision and abnormal eye movements (nystagmus) in early infancy and childhood. This disease has been associated with at least 15 different genes, and gene therapy for one of the forms, the congenital blindness disorder, LCA2, has been investigated recently in animals models and in humans [2-8].

The severe visual impairment in most patients affected by LCA, as well as other early-onset retinal degenerations, is difficult to quantify with conventional clinical instrumentation [9]. In the past, there was no need to be exceedingly quantitative [10]. Recently, preclinical success in animal models of LCA and the development of several phase 1 clinical trials of gene therapy for LCA in humans had made it worthwhile to explore clinically feasible methods that can precisely quantify the visual function of these patients. Several techniques have been used in the first three independent clinical trials of LCA2 gene therapy, which initiated nearly contemporaneously in 2007 (NCT00481546[11], NCT00516477[8], NCT00643747[12], ClinicalTrials.gov), in order to assess the improvement in visual function. Such techniques can be either subjective, that is, requiring an active response by the patients, or objective, that is, not requiring a voluntary response from the patients. As the gene therapy trials are open-label with the patients not blinded to the treatment, objective techniques have provided more reliable results. Until now, the objective ophthalmologic techniques applied in these studies have been exclusively electroretinogram and pupillometry. However, electroretinogram was unable to show the improvements achieved by gene therapy, as it was unrecordable both before and after treatment [8,11,12]. In contrast, pupillometry appeared to be a useful additional measure as it provides quantitative information in infants, in children and adults. Pupillometry consisted of the measurement of the light-induced contraction of the iris muscle due to the pupillary light reflex (PLR). The major signal input for PLR originates from rod and cone photoreceptors in the outer retina [13]. The accessibility of the iris for observation provides an easy, non-invasive, and non-contact method to explore visual function through the study of PLR. The adoption of pupillometry as a useful additional outcome measure in therapeutic trials of LCA was suggested in 2004 by Aleman who explored the feasibility of the technique to quantify the visual abnormalities in LCA patients [14]. Several studies investigated its utility for evaluation of improvement of light reflexes and asymmetry between the two eyes [15,16]. This evidence motivated the adoption of pupillometric analysis in the framework of the clinical gene therapy LCA trial registered as NCT00516477 in ClinicalTrials.gov [8]. The pupillometry was performed by using a commercial pupillometer and software. As the developed clinical protocol was unique, first a manual procedure was defined to perform the analysis but it was time-consuming. Successively, an ad hoc MATLAB package was developed in order to streamline the analysis and reduce the intervention of the operator, which may cause experimenter’s bias.

In this paper, we propose and describe the most updated version of the pupillometric analysis method which has been explored in the framework of the clinical trial NCT00516477 for evaluation of pupillary reflexes in LCA2 patients undergoing gene therapy [8].

As a case study, we reported the results of the analysis of the three Italian patients involved in the clinical trial focusing on two post-treatment time-points at 1 year and 3 years respectively.

## Methods

### Case study

Three patients (further referred here as subjects 1, 2, and 3) involved in the clinical gene therapy LCA2 trial were analysed in this study: subject 1 is a 26 year old female, subject 2 is a 26 year old male, and subject 3 is a 19 year old female [8]. All patients were affected by LCA2 without other complications. None of the subjects took drugs affecting sympathetic or parasympathetic pupillary function. They had not past history of ocular operations, non-symmetrical pupil, misshapen pupil, or other conditions affecting pupillary reflexes. After informed consent and confirmation of trial eligibility, including legal blindness, the eye with worse visual function was selected for sub-retinal gene delivery through vector AAV2-hRPE65v2. In all the three patients, the right eye was injected. Subjects were evaluated before and after treatment as described in a previous report [8]. In this study, we focused on two post-treatment time-points: 12 months and 36 months.

### Pupillometric clinical protocol

The stimulation protocol was modelled after the “swinging flashlight test”, in order to identify relative afferent pupillary defects (rAPDs) [17]. Responses were measured after 40 minutes of dark adaptation by sequential stimulation in each eye with white light under low, medium and high intensity conditions (0.04, 0.4 and 10 lux, respectively) [17]. Pupillary responses to light were recorded with variants of the basic protocol, as described also elsewhere [6,7].

Two tests were performed:

● Test 1 - eight consecutive cycles consisting of a light stimulus presented for 0.2 seconds followed by a 1 second dark interval;

● Test 2 - six consecutive cycles consisting of a light stimulus presented for 1 second followed by a 0.6 second dark interval.

In both tests, the light stimulus was presented alternatively to the right and left eye and each test was repeated twice: the first time starting with the stimulation of the right eye, the second time starting with the stimulation of the left one. Each sequence of light stimulus was interleaved with a pause of at least 3 seconds in order to recover baseline pupil diameter. The tests were usually performed in the morning, after at least 8 hours of sleep.

### Pupillometric system

Pupil responses were recorded simultaneously in both eyes with a Procyon P2000 pupillometer and PupilFit4 software (Monmouthshire, UK). Each eyepiece was equipped with infra-red diodes GaA1A’s type SFH485 with peak emission at 880 nm to illuminate the pupil without stimulating the eye, and an infra-red sensitive camera that captured the video images at 25 frame/s, allowing the pupil diameter of both eyes to be measured every 40 ms. Images were saved in bitmap format after real-time processing. Means, maximums and minimums of the pupil diameter for the entire test were computed by PupilFit4. Responses to individual stimuli were not computed. Therefore, a package of functions was developed in Matlab (The MathWorks Inc., Massachusetts, USA) to provide the following functionalities for each acquired frame:

● pupil detection;

● manual check/revision of pupil detection;

● elimination of unsuitable frames (e.g. due to blinking);

● computation of pupillometric features for each stimulus;

● statistical analysis as described below.

The offline image processing algorithm determined the pupil diameter when the results of the PupilFit4 were not reliable, that is, when there were percentage variations in the pupil diameter from previous frames higher than a user-defined threshold (10% default).

The algorithm consisted of four major steps:

● image thresholding - as the images were acquired under the same light condition, the darkness of the pupil is approximately the same for all the subjects (around 35 gray level), for that reason the threshold was set at the same value (40 gray level).

● hole filling - holes, mainly due to light reflexes, were filled using an algorithm based on morphological reconstruction [18].

● small object removing - all connected components (objects) that had fewer than a N pixels, were removed from the image. The number N was chosen in order to eliminate objects with a surface area less than 2 mm^2^, equivalent to a circle with a radius of 0.8 mm.

● edge detection - morphological operations, such as dilatation and removing the interior pixel, were performed in order to leave only the boundary pixels, which should represent the pupillary border.

● pupil detection and diameter measurement - generalized Hough transform was implemented to detect the position and the diameter of the pupil, modelled as a circular shape.

**Figure 1:**
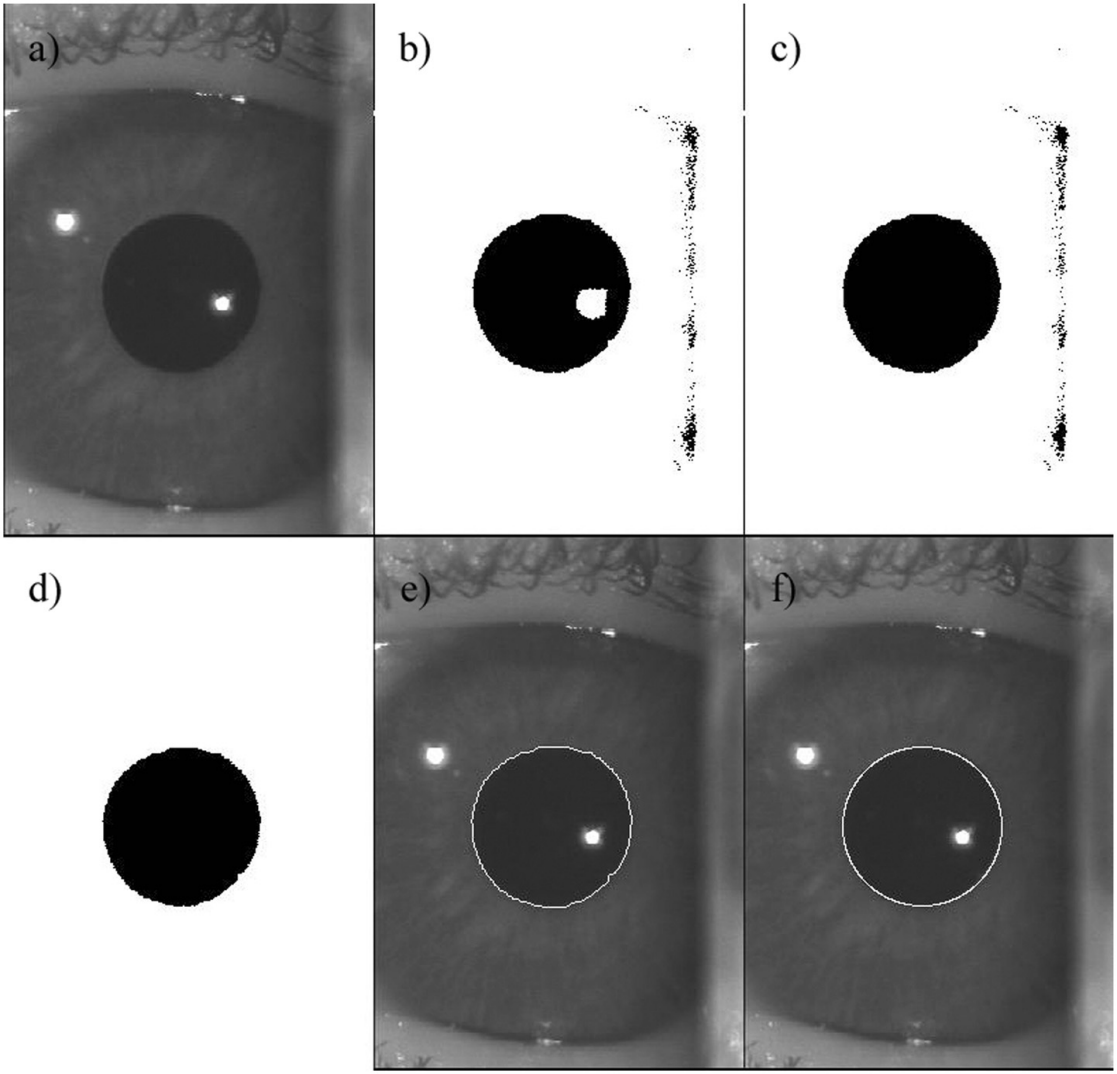
**Description of the off-line pupil detection algorithm steps.** (**a**) image acquired by the pupillometer; (**b**) image after the thresholding; (**c**) image after hole filling; (**d**) image after small object removing; (**e**) original image with the pupil border enhanced in white thanks to edge detection. (**f**) original image with the circle (in white) which fits the pupil border.

Figure 1 shows the images obtained in the major algorithm steps until the best circle that fits the pupil is found.

A user-friendly Graphical User Interface was developed to check the results of the automatic pupil detection. It shows the image of the eye and the shape of the pupil detected by the off-line processing. If the user is satisfied by the automatic pupil detection, he/she just has to click the mouse, otherwise he/she can adjust the position and diameter of the detected shape by using the mouse. If the pupil could not be detected, not even manually, for instance due to blinking, the user could evaluate whether to accept a linear interpolation of the diameter between neighbor frames not affected by noise or, in extremis, to discard the stimulus.

According to the experience of previous studies [6,7,19], pupillometric data were quantified through the following parameters:

·Baseline Diameter (BD) computed as the average of the pupil diameter 100 ms prior to the light stimulus;

·Minimum Amplitude (MA) defined as the lowest pupil diameter after a light stimulus;

Response Amplitude (RA) computed as the difference between BD and MA;

·Percent Constriction (PC) defined as the ratio between MA and BD expressed as percentage;

·Response Time (RT) defined as the difference between the time corresponding to the minimum amplitude and the time corresponding to the stimulus;

·Constriction Velocity (CV) computed as the ratio between RA and RT.

**Figure 2:**
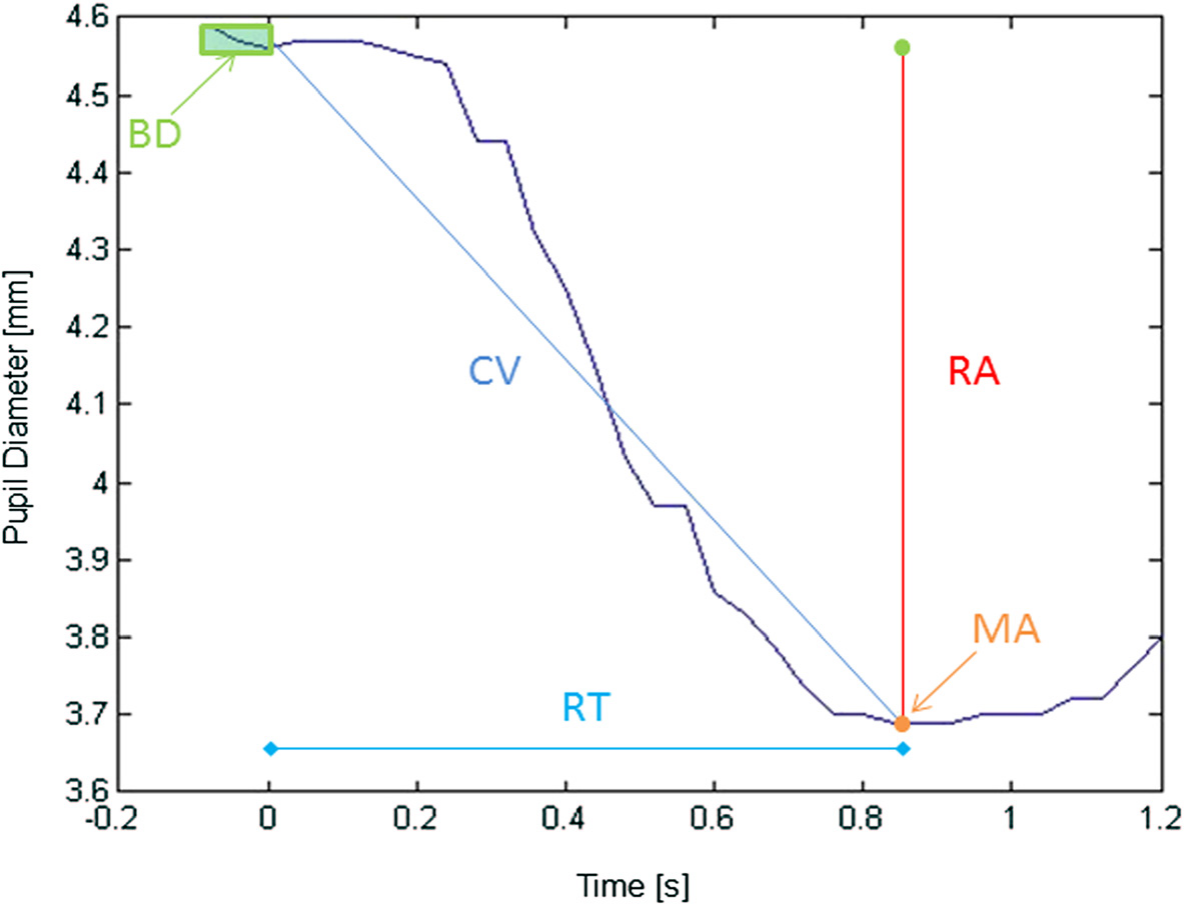
**Pupil reflex of a subject involved in the study after a 0.2 second light stimulus triggered at zero second.** The indicated parameters are computed as follows: BD, average of the pupil diameters 100 ms prior to the light stimulus (values to be averaged are in the green rectangular box): MA, the lowest pupil diameter after a light stimulus (represented by the orange point): RA, BD-MA (represented by the red segment); RT, t_minimum amplitude_- t_stimulus_ (t = time) (represented by the light blue segment): CV, RA/RT (represented by the slope of the dark blue line).

Figure 2 shows an example of pupil reflex and the definition of the parameters.

A non-parametric statistical paired test (Wilcoxon signed rank test) was performed to compare:

● the pupillometric parameters obtained when the right eye was stimulated with those obtained when the left eye was stimulated;

● the pupillometric parameters obtained at the first time-point (1 year after treatment) with those obtained at the second time-point (3 years after treatment).

## Results

**Figure 3:**
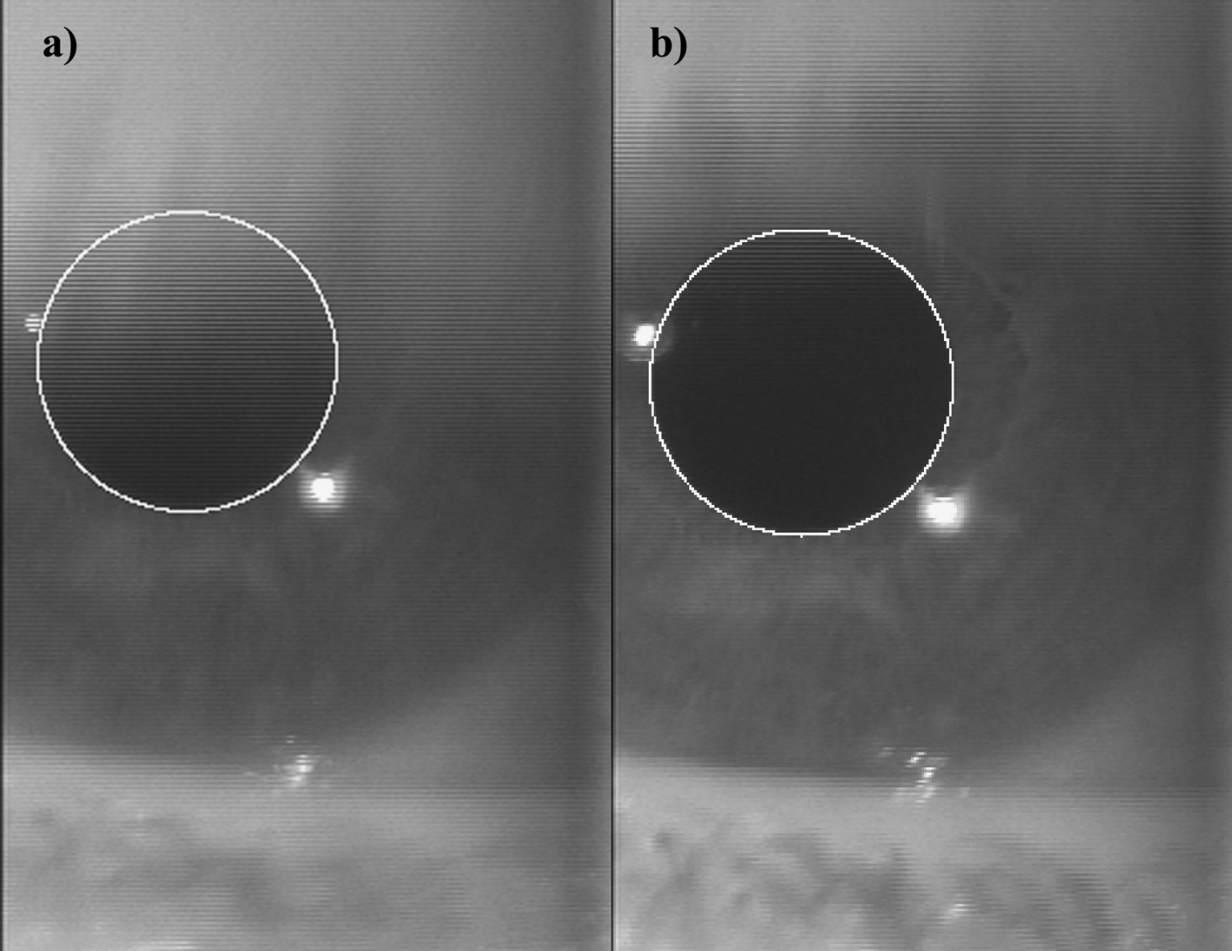
Example of pupil diameter detection when the eyelashes are over the pupil (a) and on the subsequent frame in which the pupil is completely visible (b).

The developed MATLAB package provided an automatic pupil detection, which was correct also for frames in which the pupil was not totally visible (up to 30% of area not visible), for instance when the eyelashes obscured the pupil, as shown in Figure 3. When the automatic detection is not accurate, the user can manually revise the pupil detection.

The results related to the comparison between the two eyes are reported in Tables 1, 2 and 3 for each subject with respect to Test 1.

**Table 1:** Comparison of pupillometric parameters between the two eyes in subject 1 (Test 1)

**Par.**	**1 year post-treatment**							**3 years post-treatment**						
	**Right eye**			**Left eye**				**Right eye**			**Left eye**			
	**Med**	**25**^**th**^	**75**^**th**^	**Med**	**25**^**th**^	**75**^**th**^	**P**	**Med**	**25**^**th**^	**75**^**th**^	**Med**	**25**^**th**^	**75**^**th**^	**P**
BD	4.67	4.30	4.98	4.47	3.89	4.69	**<0.01**	4.17	3.75	4.60	4.14	3.60	4.54	0.21
MA	4.35	3.78	4.57	4.37	4.03	4.70	**0.02**	4.07	3.52	4.34	4.06	3.54	4.39	0.69
RA	0.37	0.28	0.52	0.02	-0.09	0.06	**<0.01**	0.20	0.04	0.33	0.04	0.00	0.14	**0.01**
PC	0.92	0.89	0.94	1.00	0.98	1.02	**<0.01**	0.96	0.92	0.99	0.99	0.97	1.00	**0.01**
RT	0.98	0.84	1.04	0.34	0.20	0.70	**<0.01**	0.90	0.48	1.02	0.26	0.22	1.04	0.10
CV	0.39	0.31	0.51	0.05	-0.27	0.15	**<0.01**	0.25	0.04	0.44	0.06	0.00	0.19	**0.01**

Par: parameters.

Med: median.

25^th^ : 25 ^th^ percentile (first quartile)/75^th^ : 75 ^th^ percentile (third quartile).

P: p-value.

**Table 2:** Comparison of pupillometric parameters between the two eyes in subject 2 (Test 1)

**Par.**	**1 year post-treatment**							**3 years post-treatment**						
	**Right eye**			**Left eye**				**Right eye**			**Left eye**			
	**Med**	**25**^**th**^	**75**^**th**^	**Med**	**25**^**th**^	**75**^**th**^	**P**	**Med**	**25**^**th**^	**75**^**th**^	**Med**	**25**^**th**^	**75**^**th**^	**P**
BD	5.24	4.42	5.72	5.09	4.26	5.77	0.35	4.92	3.91	5.63	4.91	3.81	5.62	0.66
MA	4.84	4.04	5.62	5.08	4.16	5.57	0.57	4.71	3.67	5.50	4.71	3.76	5.52	0.33
RA	0.21	0.14	0.33	0.06	0.00	0.22	**0.02**	0.16	0.08	0.28	0.09	0.03	0.16	**0.02**
PC	0.96	0.93	0.98	0.99	0.96	1.00	**0.02**	0.97	0.93	0.98	0.98	0.97	1.00	**0.01**
RT	0.76	0.60	1.16	0.30	0.20	0.94	**0.049**	0.72	0.50	0.94	0.86	0.54	1.10	0.38
CV	0.30	0.22	0.48	0.14	-0.02	0.29	0.098	0.22	0.11	0.42	0.12	0.04	0.19	**0.03**

Par: parameters.

Med: median.

25^th^ : 25 ^th^ percentile (first quartile)/75^th^ : 75 ^th^ percentile (third quartile).

P: p-value.

**Table 3:** Comparison of pupillometric parameters between the two eyes in subject 3 (Test 1)

**Par.**	**1 year post-treatment**							**3 years post-treatment**						
	**Right eye**			**Left eye**				**Right eye**			**Left eye**			
	**Med**	**25**^**th**^	**75**^**th**^	**Med**	**25**^**th**^	**75**^**th**^	**P**	**Med**	**25**^**th**^	**75**^**th**^	**Med**	**25**^**th**^	**75**^**th**^	**P**
BD	4.00	3.84	4.03	3.75	3.56	4.00	0.07	3.85	3.64	4.42	3.74	3.61	4.32	0.88
MA	3.52	3.38	3.63	3.70	3.47	3.78	**0.03**	3.65	3.47	4.01	3.75	3.44	4.14	0.64
RA	0.39	0.29	0.51	0.05	-0.03	0.23	**<0.01**	0.20	0.14	0.33	0.08	-0.02	0.23	0.10
PC	0.90	0.87	0.92	0.98	0.94	1.01	**<0.01**	0.94	0.92	0.96	0.98	0.94	1.01	0.10
RT	0.72	0.56	0.94	0.52	0.20	0.68	**<0.01**	0.66	0.52	0.90	0.66	0.60	0.80	0.78
CV	0.63	0.47	0.69	0.12	-0.09	0.34	**<0.01**	0.33	0.21	0.51	0.13	-0.06	0.36	0.11

Par: parameters.

Med: median.

25^th^ : 25 ^th^ percentile (first quartile)/75^th^ : 75 ^th^ percentile (third quartile).

P: p-value.

At 1 year follow-up, Response Amplitude (RA) and Constriction Velocity (CV) were higher when the injected (right) eye was stimulated compared to when the uninjected (left) eye was stimulated. These differences were statistically significant in all three subjects (with the exception of CV in patient 2). The asymmetry between left and right eye persisted through 3 years, the latest time-point tested, even if the differences of RA were statistically significant only in subjects 1 and 2 and the differences of CV were statistically significant only in subject 1. In Test 2, RA and CV were higher when the injected (right) eye was stimulated instead of the uninjected (left) eye at both follow-up time-points, even if the differences were not statistically significant. Results of Test 2 are not reported in details as no statistical difference was found for any parameter.

The results of the comparison between the two follow-up time-points were reported in Tables 4, 5 and 6 for each subject. No significant difference in RA, PC, RT, and CV between 1-year and 3-year time-points was apparent when data from both eyes were considered (neither Test 1 nor Test 2). Significant differences were found in BD and MA for subjects 1 and 2.

**Table 4:** Comparison of pupillometric parameters between the two follow-up time-points in subject 1 (Both Tests)

**Par.**	**Test 1**							**Test 2**						
	**1 year time-point**			**3 year time-point**				**1 year time-point**			**3 year time-point**			
	**Med**	**25**^**th**^	**75**^**th**^	**Med**	**25**^**th**^	**75**^**th**^	**P**	**Med**	**25**^**th**^	**75**^**th**^	**Med**	**25**^**th**^	**75**^**th**^	**P**
BD	4.56	4.20	4.77	4.14	3.71	4.59	**<0.01**	4.56	4.00	4.81	4.03	3.93	4.24	**<0.01**
MA	4.37	3.91	4.60	4.06	3.52	4.38	**<0.01**	4.22	3.67	4.73	3.95	3.70	4.06	**<0.01**
RA	0.16	0.01	0.39	0.06	0.01	0.25	0.06	0.16	0.06	0.29	0.16	0.05	0.28	0.26
PC	0.96	0.92	1.00	0.99	0.94	1.00	0.11	0.97	0.93	0.99	0.96	0.93	0.99	0.42
-RT	0.74	0.28	1.00	0.80	0.24	1.04	0.77	0.94	0.68	1.22	1.00	0.48	1.24	0.77
CV	0.26	0.02	0.42	0.13	0.04	0.30	0.30	0.14	0.07	0.33	0.18	0.05	0.31	0.48

Par: parameters.

Med: median.

25^th^ : 25 ^th^ percentile (first quartile)/75^th^ : 75 ^th^ percentile (third quartile).

P: p-value.

**Table 5:** Comparison of pupillometric parameters between the two follow-up time-points in subject 2 (Both Tests)

**Par.**	**Test 1**							**Test 2**						
	**1 year time-point**			**3 year time-point**				**1 year time-point**			**3 year time-point**			
	**Med**	**25**^**th**^	**75**^**th**^	**Med**	**25**^**th**^	**75**^**th**^	**P**	**Med**	**25**^**th**^	**75**^**th**^	**Med**	**25**^**th**^	**75**^**th**^	**P**
BD	5.15	4.30	5.77	4.92	3.82	5.62	**<0.01**	4.99	4.26	5.23	4.67	4.19	5.13	0.55
MA	5.05	4.11	5.57	4.71	3.71	5.50	**<0.01**	4.65	4.05	4.97	4.36	3.88	4.90	0.48
RA	0.17	0.06	0.28	0.11	0.04	0.22	0.24	0.24	0.05	0.34	0.24	0.05	0.43	0.53
PC	0.97	0.95	0.99	0.97	0.96	0.99	0.68	0.95	0.93	0.99	0.95	0.90	0.99	0.54
RT	0.62	0.20	1.06	0.72	0.52	1.08	0.30	1.04	0.56	1.50	1.10	0.70	1.34	0.80
CV	0.26	0.13	0.34	0.15	0.07	0.26	0.38	0.20	0.09	0.34	0.21	0.05	0.44	0.89

Par: parameters.

Med: median.

25^th^ : 25 ^th^ percentile (first quartile)/75^th^ : 75 ^th^ percentile (third quartile).

P: p-value.

**Table 6:** Comparison of pupillometric parameters between the two follow-up time-points in subject 3 (Both Tests)

**Par.**	**Test 1**							**Test 2**						
	**1 year time-point**			**3 year time-point**				**1 year time-point**			**3 year time-point**			
	**Med**	**25**^**th**^	**75**^**th**^	**Med**	**25**^**th**^	**75**^**th**^	**P**	**Med**	**25**^**th**^	**75**^**th**^	**Med**	**25**^**th**^	**75**^**th**^	**P**
BD	3.98	3.69	4.02	3.79	3.64	4.35	0.66	3.65	3.41	4.01	3.97	3.39	4.22	0.32
MA	3.55	3.40	3.72	3.67	3.47	4.08	0.08	3.38	3.09	3.62	3.49	3.03	3.96	**0.03**
RA	0.23	0.05	0.46	0.16	0.03	0.24	0.15	0.32	0.12	0.62	0.30	0.19	0.44	0.57
PC	0.94	0.89	0.98	0.96	0.94	0.99	0.12	0.91	0.84	0.96	0.92	0.89	0.95	0.57
RT	0.62	0.48	0.82	0.66	0.58	0.84	0.24	0.90	0.66	1.18	0.98	0.64	1.12	0.96
CV	0.38	0.10	0.65	0.22	0.04	0.46	0.11	0.33	0.13	0.66	0.32	0.22	0.42	0.67

Par: parameters.

Med: median.

25^th^ : 25 ^th^ percentile (first quartile)/75^th^ : 75 ^th^ percentile (third quartile).

P: p-value.

Moreover, the analysis was performed also by distinguishing PLR according to the light intensity stimulus. The asymmetry between the two eyes with respect to RA, was statically significant in subject 1 at all three intensity levels, in subject 2 only at the highest one, in subject 3 only at the lower one.

## Discussion

In this study, we describe the application of pupillometric analysis for evaluation of pupillary reflexes in patients undergoing gene therapy. This is clinically relevant because the pupillometry appeared to be the only objective ophthalmologic test able to detect an amelioration of visual function in treated LCA patients. The results showed that the patients had a higher PLR when the treated eye was stimulated rather than when the control one was stimulated. These findings are especially noteworthy as before treatment the treated eye had the worst responses [8]. The absence of statistical differences between the two selected time-points suggested stability over time of therapeutic effect mediated by gene delivery. These results were consistent with the improvement and the stability over time of other clinical parameters such as best corrected visual acuity [8]. As expected according to Kawasaki [20], Test 1, based on short stimulus cycles and dark intervals, provided significant results compared to Test 2. The choice of presenting different intensity light stimulus was motivated by the findings by Bergamin [15], who concluded that the ability to detect asymmetry between the two eyes was best obtained by testing over a range of light intensity. For instance, if only the middle light intensity level had been adopted in this study, no significant differences would have been found in subjects 2 and 3. Moreover, results presented in this paper confirm the usefulness of pupillometry to assess effect of intervention in LCA patients.

Pupillometry has been widely investigated in patients with diabetes to provide information about autonomic neuropathy [21-24], showing that the baseline pupil size, diameter and time parameters during the contraction phase are mainly under sympathetic control while both sympathetic and para-sympathetic systems are active during the recovery phase. Since the selected patients showed no evidence of autonomic dysfunction, and the aim of the current study was not to show relationship between PLR parameters and autonomous nervous system (ANS), we focused on parameters derived from the contraction phase of the pupil reflex which, according to the findings by Bergamin [15], are more useful than those extracted from the dilatation phase to show asymmetry between the two eyes. In particular, RA appears to be the most useful parameter to show this asymmetry, consistently with the results of previous reports in the framework of the same clinical trial [6-8]. Obviously, PC, which is the ratio of RA and BD, provides the same information as RA. In contrast, in our patients BD appeared to be insensitive to measure visual impairment, coherently with the findings by Aleman [10], who observed near-normal pupil diameters under dark-adapted conditions in a cohort of 18 patients with LCA, compared to 8 healthy subjects.

The comparison of our results with the findings of other clinical gene therapy trial using pupillometric analysis [25] is limited by the differences in the adopted protocol [26]. Cideciyan [25] computed RA as the difference between BD and the pupil diameter at a fixed time after the onset of the stimulus and derived the so-called luminance-response functions from RA to stimulus of increasing intensities (over a ∼ 9 log unit range). Cideciyan [25] reported that two of the three patients showed a shift of their luminance-response functions to lower stimulus intensities in their injected eye, signifying a better sensitivity, while there was no change in the control eyes in the three patients nor in the injected eye of a third patient. We underline that, in the current study and in the related clinical trial, a different approach was adopted based on binocular pupillometry method designed to detect relative afferent pupillary defects [17]. This method enabled to show significant improvement in PLR in all the patients after treatment, as previously reported [6-8]. Although the underlined differences, in both trials, transient/dynamic (as opposed to steady state/static) PLR elicited by short duration stimuli was adopted as suggested by Aleman [10,14]. The results of both the trials confirm the feasibility and usefulness of dynamic pupillometry as an objective and non-invasive measure of the visual functionality in LCA patients, as also concluded by a recent conference abstract by Kawasaki [27].

As regards software implementation, the developed MATLAB package improved the on-line pupil detection and automatically computed the pupillometric parameters for each stimulus. We believe that a check by the user is required in this field, as it would be improper that a result derived from an incorrect automatic detection might affect the decision whether or not an expensive therapy should be further investigated or transferred to clinical practice. A manual standardized protocol had been developed to check the results of the online pupil detection but the procedure required about 60 minutes for each pupillometric time-series. For that reason, a Grafical User Interface was developed to enable a semi-automatic revision of computerized detection, eliminating the need of this time-consuming procedure in which the user should manually check frame by frame using other imaging software. We tested that the same results (particularly, in terms of frames identified because of inaccurate online pupil detection) were achieved but in about 5 minutes (versus about 60 minutes of the manual procedure) for each pupillometric time-series.

Other objective techniques, such as fMRI, have been investigated to provide objective information about the improvement in retinal and visual function of LCA2 patients treated by gene therapy [5]. fMRI has been shown to give useful information about visual cortex activity but it is a much more expensive technology and is not suitable for all patients, as some patients were unable (or not willing) to undergo MRI because of a medical contra-indication (for example, a prosthesis), claustrophobia, non-collaboration.

This study presented some limitations, which are described in the following paragraphs. The implemented algorithm is designed to improve the real-time processing results provided by PupilFil4, and is not intended to be a method for real-time pupil measurement, such as those proposed by Zhu [28] or by Iskander [29]. The number of pupillometric parameters to be computed could be expanded, for instance the implementation of iris detection could be a further development in order to compute parameters based on the pupil-iris radius ratio as introduced by Fotiou [30]. However, the choice of the parameters was driven by the unique situation of severely impaired patients, who were legally blind before treatment. Another limitation is the small sample size, but it is typical of a phase 1 clinical trial in this field, as it is very hard to recruit a large number of patients with the selected mutation since LCA2 is a rare disease. Five Italian patients were recruited thanks to the national relevance of the ophthalmologic clinic in Naples, and, at the moment, data of long-term follow-up are available for these three patients.

An interesting further development could extend to using pupillometric data as a measure of the ANS activity, for instance in conjunction with the Heart Rate Variability, another non-invasive marker of the ANS activity, widely investigated by the authors (PM and LP) both in healthy subjects [31] and in patients [32-34].

## Conclusions

In conclusion, the results shown in this paper confirm that pupillometry is a useful objective measure to assess the effect of gene therapy in LCA patients. The developed program streamlined the analyses and allowed rapid statistical analysis of a range of parameters associated with PLR. The methods could be suitable in randomized clinical trials in order to assess the efficacy of gene therapy in LCA patients with no/low risk of patients’ and experimenters’ bias. With reference to the case study, the pupillometric analysis provided further support for the persistence over the time (3 years) of a significant improvement of retinal/visual function in the treated eyes.

## Competing interests

The authors declare that they have no competing interests.

## Authors’ contributions

PM conceived the study and developed the method for off-line processing and data analysis, LP revised them. JB provided the set-up of the hardware for the pupillometry measurement. FT, SR, JB and FS designed the clinical protocol. FT performed the pupillometric test and acted as user of the MATLAB software. PM drafted the manuscript, LP, FT, SR and JB participated to drafting of the manuscript. FT, SR and FS recruited the subjects. All authors read, reviewed and approved the final manuscript.
